# Association between respiratory tract diseases and secondhand smoke exposure among never smoking flight attendants: a cross-sectional survey

**DOI:** 10.1186/1476-069X-6-28

**Published:** 2007-09-26

**Authors:** Jon O Ebbert, Ivana T Croghan, Darrell R Schroeder, Judith Murawski, Richard D Hurt

**Affiliations:** 1 Mayo Clinic College of Medicine, Mayo Clinic, 200 First Street SW, Rochester, MN 55905, USA; 2 Association of Flight Attendants-CWA, AFL-CIO, Air Safety, Health, & Security Department, 501 3rd St. NW #200, Washington, DC 20001, USA

## Abstract

**Background:**

Little is known about long-term adverse health consequences experienced by flight attendants exposed to secondhand smoke (SHS) during the time smoking was allowed on airplanes. We undertook this study to evaluate the association between accumulated flight time in smoky airplane cabins and respiratory tract diseases in a cohort of never smoking flight attendants.

**Methods:**

We conducted a mailed survey in a cohort of flight attendants. Of 15,000 mailed questionnaires, 2053 (14%) were completed and returned. We excluded respondents with a personal history of smoking (n = 748) and non smokers with a history of respiratory tract diseases before the age of 18 years (n = 298). The remaining 1007 respondents form the study sample.

**Results:**

The overall study sample was predominantly white (86%) and female (89%), with a mean age of 54 years. Overall, 69.7% of the respondents were diagnosed with at least one respiratory tract disease. Among these respondents, 43.4% reported a diagnosis of sinusitis, 40.3% allergies, 30.8% bronchitis, 23.2% middle ear infections, 13.6% asthma, 13.4% hay fever, 12.5% pneumonia, and 2.0% chronic obstructive pulmonary disease. More hours in a smoky cabin were observed to be significantly associated with sinusitis (OR = 1.21; p = 0.024), middle ear infections (OR = 1.30; p = 0.006), and asthma (OR = 1.26; p = 0.042).

**Conclusion:**

We observed a significant association between hours of smoky cabin exposure and self-reported reported sinusitis, middle ear infections, and asthma. Our findings suggest a dose-response between duration of SHS exposure and diseases of the respiratory tract. Our findings add additional evidence to the growing body of knowledge supporting the need for widespread implementation of clean indoor air policies to decrease the risk of adverse health consequences experienced by never smokers exposed to SHS.

## Background

According to United States Surgeon General's Report, available evidence suggests a causal relationship between secondhand smoke (SHS) exposure and tobacco-related diseases [1]. In healthy adults, SHS is associated with upper and lower respiratory tract diseases and an increased risk of asthma and chronic obstructive pulmonary disease (i.e., bronchitis and emphysema). Among lifetime nonsmokers, SHS is also associated with an increased risk of lung cancer and death and disability from coronary heart disease (CHD). Pooled analyses suggest an overall 20–30% increase in the risk of lung cancer and CHD attributable to SHS.

Prior to 1999, by which time 97% of flights to and from the United States were smoke-free, flight attendants (FAs) experienced 6 to 7 times the SHS exposure of ground-based workers and 14 times that of the average person [2]. Based upon surveys conducted of general flight experiences, FAs experienced a variety of physical symptoms ranging from respiratory complaints to fatigue which may have been attributable to a variety of in-flight factors in addition to SHS before the airlines became smoke-free [3]. However, in a study conducted before and after in-flight smoking was eliminated, a causal link was established between SHS and ocular symptoms, decreased tear-film stability, and alterations in nasal patency [4]. Less is known about the potential long-term health consequences experienced by FAs exposed to SHS during the time smoking was allowed on airplanes.

A number of studies have obtained FA personal health histories in order to assess the health and comfort of FAs in airliner cabins [3]. Most of these have focused on short-term effects and respiratory symptoms, but also included a mixture of flight attendants who were smokers and non smokers. None of the published studies have evaluated the association between the accumulated flying time and diseases of the respiratory tract. We undertook the present study to evaluate the association between accumulated flight time prior to the airlines becoming smoke-free and reported medical conditions in a large cohort of FAs who were never smokers.

## Methods

### Survey development

We developed a 26-item, self-administered mailed questionnaire. Items on the survey ascertained demographics, smoking history, personal and family medical history, exposure to toxic chemicals, alcohol consumption, and duration of exposure to an airline cabin prior to institution of smoke-free airlines. All items were direct and face-valid as determined by the investigators and structured based upon previous population surveys [5]. We conducted readability and usability testing of the survey on a sample of 30 volunteers who were active or retired FAs. The volunteers were recruited by mail. Feedback by these volunteers on the survey was provided to investigators in the written form which was collated and incorporated into the final survey.

### Survey sample

The study was reviewed and approved by the Mayo Institutional Review Board (IRB) and informed consent was obtained from all participants. Subjects were identified from a member database maintained by the Association of Flight Attendants (AFA). Current and past flight attendants (FAs) with pre-1987 seniority were selected to be surveyed. The AFA did not have permission from its members to provide a mailing list with their identifiers to an outside organization, thus, the initial contact with potential subjects was made through the AFA mailing house. No identifiable information was provided to the Mayo investigators prior to the subjects signing informed consent and completing the survey.

### Survey procedure

In January 2005, approximately 15,000 surveys were mailed from the AFA mailing house following the mailing of an introductory letter. The introductory letter was from the president of the AFA. This letter explained the background of the study, the collaboration between AFA and Mayo Clinic, and encouragement to participate. The letter informed members of the cohort that this study would examine the association between SHS and adverse health outcomes but not specifically about respiratory tract diseases. The second mailing included more detail about the study, the survey, and a consent form. In order to protect confidentiality, surveys were mailed by the AFA mailing house using the AFA mailing house identifier on the envelope. Completed surveys were mailed back to the Mayo Clinic Survey Research Center for verification of consent and data entry. A single mailing was conducted with no follow-up calls.

### Survey items

Medical diagnoses were assessed by asking if respondents "have ever taken medication for, had surgery for, or been told by a doctor" that they have the condition of interest. If respondents indicated affirmatively, they were asked to indicate the age at which they were diagnosed with the condition.

### Response rate

Of the 15,000 questionnaires provided to the AFA mailing house, 2053 (14%) were completed and returned to the Mayo Clinic Survey Research Center. Information on the number of undeliverable survey packets due to incorrect or outdated address information was unavailable.

We were specifically interested in whether or not exposure to SHS increased the risk for the development of respiratory tract disease. Therefore, we excluded respondents who had a personal history of smoking (n = 748) or non smokers with a history of respiratory tract disease before the age of 18 years (n = 298). The remaining 1007 respondents were never smokers with no reported respiratory tract disease prior to the age of 18 years and formed the study sample for the current report.

### Statistical methods

As part of the survey, respondents recorded information regarding the number of hours they spent flying during the time that smoking was allowed in airplane cabins. Since mandated smoke-free policies changed over time and some carriers adopted voluntary smoke-free policies prior to these dates, respondents recorded information separately for each carrier (i.e., company) for which they worked during the time that smoking was allowed. Using these data, the cumulative number of hours spent flying in a smoky cabin was calculated for each respondent.

Logistic regression analyses were performed to assess whether the diagnosis of disease after the age of 18 was associated with the duration of time spent in a smoky cabin. For these analyses, the duration of time spent in a smoky cabin was analyzed as both a continuous variable and also as a categorical variable with categories defined using quartiles of the observed distribution (≤11,030 hours; 11,031 to 18,240 hours; 18,241 to 27,750 hours; and ≥27,751 hours). The respondent's age at the time the survey was included as a covariate in all models. Since respondent age was missing for approximately 5% of the study sample, the logistic regression analyses were performed using a multiple imputation approach (10 imputed datasets constructed using Markov Chain Monte Carlo method) with parameter estimates adjusted using Rubin's rules [6]. Analyses were also performed using the subset of respondents with complete data to ensure that findings were consistent.

Separate analyses were performed for each disease of interest. Findings from the logistic regression analyses are summarized using odds ratios (ORs) and corresponding 95% confidence intervals (CIs). For the analysis with time spent in the smoky cabin treated as a categorical variable, the ORs are calculated using the lower quartile group as the reference group. For the analysis with time spent in the smoky cabin treated as a continuous variable, the OR was calculated for an increase of 16,720 hours which corresponded to the difference between the 25^th ^and 75^th ^percentile of the observed distribution. Respondent characteristics (e.g. age, gender, race) were compared across quartile groups using the Kruskall-Wallis test for continuous variables and the chi-square test for categorical variables. All analyses were performed using SAS (version 8.2; SAS Institute, Inc, Cary, NC). In all cases, two-tailed p-values ≤ 0.05 were considered statistically significant.

## Results

### Sample demographics

The overall study sample was predominantly white (86%) and female (89%), with a mean (SD) age of 53.5 (6.2) years (Table 1). The median [interquartile range (IQR)] number of hours spent in a smoky cabin was 18,240 (11,030 to 27,750) hours. Comparing across quartile groups defined based on the distribution of time spent in a smoky cabin, significant differences were found with respect to age at time of the survey (p < 0.001), age at the time of first job with any airline (p < 0.001), and gender (p = 0.017). In all cases, the direction of the observed differences was consistent with what would be expected for this study population. Specifically, respondents who reported more time in the smoky cabin tended to be older at the time of the survey, younger when they started working for the airline industry, and female.

**Table 1:** Characteristics of respondents in a survey of flight attendants and secondhand smoke exposure

Quartiles of Total Hours Spent in a Smoky Cabin		Quartiles of Total Hours Spent in a Smoky Cabin			
Characteristic	Overall (N = 1007)	Q1 (n = 252)	Q2 (n = 257)	Q3 (n = 246)	Q4 (n = 252)

Age at time of survey, years					
mean (SD)	53.5 (6.2)	50.3 (6.6)	52.7 (5.8)	54.8 (5.2)	56.6 (5.2)
median (IQR)	54 (49 – 58)	49 (45 – 55)	54 (48 – 57)	55 (51 – 59)	57 (54 – 60)
Age at time of first airline job, years					
mean (SD)	22.7 (3.4)	23.8 (3.7)	22.6 (2.8)	22.5 (3.3)	21.8 (3.3)
median (IQR)	22 (20 – 24)	23 (21 – 25)	22 (21 – 24)	22 (20 – 23.5)	21 (20 – 23)
Total hours spent in smoky cabin, hr					
mean (SD)	20293 (13,219)	5946 (2,929)	14812 (1,996)	22782 (2,634)	37802 (11,466)
Median (IQR)	18240 (11,030 – 27,750)	5922 (3,600 – 8,424)	15048 (13,200 – 16,311)	22840 (20,580 – 25,160)	33600 (30,624 – 40,800)
Gender, n (%)					
Male	110 (11)	38 (15.2)	30 (11.7)	26 (10.6)	16 (6.4)
Female	893 (89)	212 (84.8)	226 (88.3)	220 (89.4)	235 (93.6)
Race, n (%)					
American Indian/Alaska Native	5 (0.5)	1 (0.4)	0(0)	2 (0.8)	2 (0.8)
Asian	38 (3.9)	12 (4.9)	8 (3.2)	9 (3.7)	9 (3.7)
Native Hawaiian/Pacific Islander	6 (0.6)	2 (0.8)	2 (0.8)	1 (0.4)	1 (0.4)
Black or African American	46 (4.7)	10 (21.7)	14 (5.5)	14 (5.8)	8 (3.3)
White	851 (86.2)	212 (85.8)	218 (85.8)	202 (83.1)	219 (90.1)
Hispanic	33 (3.3)	9 (3.6)	9 (3.5)	13 (5.4)	2 (0.8)
Multiple/Other	8 (0.8)	1 (0.4)	3 (1.2)	2 (0.8)	2 (0.8)

SD = standard deviation; IQR = interquartile range

### Frequency of respiratory tract disease and association with time spent in smoky cabin

Overall, 69.7% of the respondents reported being diagnosed with at least one respiratory tract disease after the age of 18 years. The overall frequency of diagnosis after the age of 18 for the individual diseases of interest included: sinusitis (43.4%), allergies (40.3%), bronchitis (30.8%), middle ear infections (23.2%), asthma (13.6%), hay fever (13.4%), pneumonia (12.5%), and chronic obstructive pulmonary disease (2.0%) (Table 2).

**Table 2:** Frequency of respiratory tract diseases in flight attendants exposed to secondhand smoke

		Quartiles of Total Hours Spent in Smoky Cabin			
Respiratory Disease	Overall, N = 1007 n (%)	Q1, N = 252 n (%)	Q2, N = 257 n (%)	Q3, N = 246 n (%)	Q4, N = 252 n (%)

Any respiratory disease	702 (69.7)	167 (66.3)	173 (67.3)	168 (68.0)	194 (77.3)
Sinusitis	437 (43.4)	103 (40.9)	105 (40.9)	116 (47.0)	113 (45.0)
Allergies	406 (40.3)	100 (39.7)	98 (38.1)	106 (42.9)	102 (40.6)
Bronchitis	310 (30.8)	61 (24.2)	83 (32.3)	82 (33.2)	84 (33.5)
Middle Ear Infections	234 (23.2)	43 (17.1)	48 (18.7)	70 (28.3)	73 (29.1)
Asthma	137 (13.6)	25 (9.9)	32 (12.5)	40 (16.2)	40 (15.9)
Hay Fever	135 (13.4)	29 (11.5)	40 (15.6)	37 (15.0)	29 (11.6)
Pneumonia	126 (12.5)	24 (9.5)	26 (10.1)	34 (13.8)	42 (16.7)
COPD	20 (2.0)	4 (1.6)	4 (1.6)	5 (2.0)	7 (2.8)

**Figure 1:**
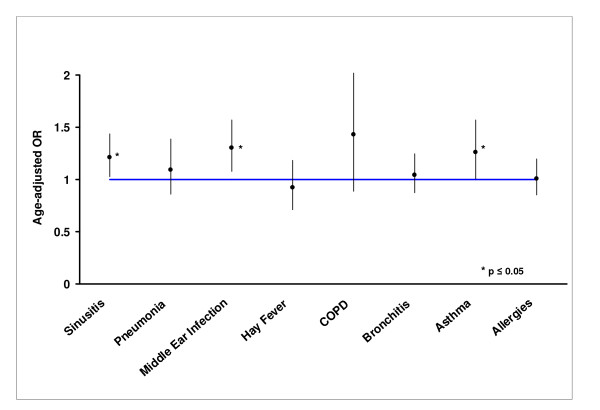
Association between total hours in a smoky airplane cabin and respiratory tract diseases. The values presented correspond to the age-adjusted odds ratio (OR) and corresponding 95% confidence interval. The OR is calculated for an increase of 16,720 hours which corresponds to the difference between the 25^th ^and 75^th ^percentile of the observed distribution.

When time spent in a smoky cabin is treated as a continuous variable, more hours in the smoky cabin was found to be significantly associated with sinusitis (OR = 1.21, p = 0.024), middle ear infections (OR = 1.30, p = 0.006), and asthma (OR = 1.26, p = 0.042) (Table 3 & Figure 1).

**Table 3:** Association of respiratory tract diseases with the duration of time spent in a smoky cabin*

		Quartiles of Total Hours Spent in Smoky Cabin			
Disease	Overall	Q1	Q2	Q3	Q4

Any respiratory disease					
Odds Ratio	1.17	1.0	1.02	1.03	1.62^†^
(95% C. I.)	(0.97, 1.42)		(0.70, 1.49)	(0.70, 1.52)	(1.06, 2.48)
Sinusitis					
Odds Ratio	1.21^†^	1.0	1.09	1.50^†^	1.47^†^
(95% C. I.)	(1.03, 1.44)		(0.76, 1.56)	(1.04, 2.18)	(1.00, 2.16)
Allergies					
Odds Ratio	1.01	1.0	0.93	1.13	1.03
(95% C. I.)	(0.85, 1.20)		(0.65, 1.34)	(0.78, 1.64)	(0.70, 1.5)
Bronchitis					
Odds Ratio	1.04	1.0	1.41	1.40	1.36
(95% C. I.)	(0.87, 1.25)		(0.95, 2.09)	(0.93, 2.10)	(0.89, 2.06)
Middle Ear Infections					
Odds Ratio	1.30^†^	1.0	1.16	2.08^†^	2.22^†^
(95% C. I.)	(1.08, 1.57)		(0.74, 1.84)	(1.33, 3.25)	(1.40, 3.52)
Asthma					
Odds Ratio	1.26^†^	1.0	1.20	1.54	1.43
(95% C. I.)	(1.01, 1.57)		(0.69, 2.11)	(0.89, 2.67)	(0.81, 2.52)
Hay Fever					
Odds Ratio	0.92	1.0	1.35	1.24	0.89
(95% C. I.)	(0.71, 1.18)		(0.81, 2.27)	(0.72, 2.13)	(0.50, 1.59)
Pneumonia					
Odds Ratio	1.09	1.0	1.02	1.40	1.71
(95% C. I.)	(0.86, 1.39)		(0.57, 1.85)	(0.79, 2.48)	(0.96, 3.03)
COPD					
Odds Ratio	1.43	1.0	0.91	1.12	1.46
(95% C. I.)	(0.89, 2.32)		(0.22, 3.72)	(0.29, 4.37)	(0.39, 5.51)

* To assess whether the diagnosis of disease after the age of 18 years was associated with the duration of time spent in a smoky cabin a series of logistic regression analyses were performed. An overall analysis was performed with the duration of time spent in a smoky cabin analyzed as a continuous variable and a supplemental analysis was performed with duration of time spent in a smoky cabin analyzed as a categorical variable with categories defined using quartiles of the observed distribution (≤11030 hours, 11031 to 18240 hours, 18241 to 27750 hours, and ≥27751 hours). The respondent's age at the time the survey was included as a covariate in all models. Findings from the logistic regression analyses are summarized using odds ratios (ORs) and corresponding 95% confidence intervals (CIs). For the analysis with time spent in the smoky cabin treated as a continuous variable, the OR is calculated for an increase of 16,720 hours which corresponds to the difference between the 25^th ^and 75^th ^percentile of the observed distribution. For the analysis with time spent in the smoky cabin treated as a categorical variable, the ORs are calculated using the lower quartile group as the reference group. † p ≤ 0.05

Time spent in a smoky cabin was also analyzed categorically using quartiles of the observed distribution. With the lowest quartile as the reference group, the diagnosis of any respiratory tract disease was increased for those in the 4^th ^quartile (OR = 1.62, p = 0.025), sinusitis was increased for both the 3^rd ^(OR = 1.50, p = 0.032) and 4^th^(OR = 1.47, p = 0.048) quartiles, and middle ear infection was increased for both the 3^rd ^(OR = 2.08, p = 0.001) and 4^th ^(OR = 2.22, p < 0.001) quartiles (Table 3).

## Discussion

We observed a significant association between total hours of smoky cabin exposure and reported sinusitis and middle ear infections among never smoking FAs who worked in the airline industry when smoking was allowed on airplanes. Our findings also suggest an increase in the odds of the development of asthma among FAs with increased exposure to smoky airplane cabins.

Previous surveys of cabin crews have consistently shown high rates of upper airway symptoms but have been focused on short-term effects [3]. SHS has been observed to be independently, positively associated with respiratory tract symptoms such as sneezing, sore throat, and cough [7] as well as nasal rhinitis [8]. The association of SHS exposure and sinusitis has not been extensively studied in adults and most of the evidence concerning the association of SHS with middle ear infections comes from the pediatric literature [1]. However, a pathophysiologic basis for causality exists as SHS causes inflammation in the nasal mucosa, and non smokers may have a heightened sensitivity to SHS exposure [9]. Unfortunately and expectedly, research on the adverse health consequences associated with SHS exposure has been hindered by tobacco industry tactics to undermine United States regulatory agencies [10].

We observed a significant association between smoky cabin exposure and asthma in our cohort of FAs, consistent with previous literature. SHS exposure has been associated with asthma in adults, and available data suggest that workplace exposure may be more detrimental than domestic exposure [11]. In a cross-sectional study of 2195 never smoking Italian women, SHS exposure from spouse and work was significantly and positively associated with an asthma diagnosis or symptoms (OR 1.50; 95% CI 1.09–2.08) [12]. In a cross-sectional study of 4197 never smoking adults in Switzerland, SHS was significantly associated with physician-diagnosed asthma (OR 1.39; 95% CI: 1.04–1.86) [13]. In a cross-sectional study of 6817 adult never smokers in Estonia, SHS exposure outside the home was associated with physician-diagnosed asthma (OR 1.79; 95% CI: 1.02–3.16), and SHS outside the home was strongly related to all respiratory symptoms in a dose-response manner [14]. In a nested-case control study of adult-onset asthma, an increased odds for the development of asthma was associated with SHS (OR 2.4; 95%, CI 1.4–4.1) [15]. In a more recent cross-sectional study of 73,605 adults in India, individuals exposed to SHS were more likely to have a diagnosis of asthma compared to non-exposed individuals (OR 1.22; 95% CI 1.08–1.38) [16]. In a prospective study of 3914 adult non smokers, SHS was significantly associated with the development of asthma over a 10-year period [relative risk (RR) = 1.45; 95% CI: 1.21–1.75] [17]. The extant literature and our study suggest a dose-response relationship between SHS exposure and adult-onset asthma.

The main strength of our study was that the survey was conducted in a large sample of FAs who were never smokers with a median of greater than 18,000 hours of SHS exposure in smoky cabins.

Our study has several limitations. First, the response rate to our survey was 14%. Because we did not have access to the mailing list, we did not have complete information regarding the accuracy of the address information and, therefore, could not determine the total population who received the survey. We were also unable to perform the standard survey methodology of second and third mailings followed up by telephone calls which would have increased our response rate. Nonetheless, our response rate was similar to the 17% response rate of the only other survey of FAs larger than ours (N = 3,412) [18]. While our sample of flight attendants is the second largest reported to date, it is the largest that reports on a subset of never smokers. Given the low response rate and partial blinding to study hypotheses, we cannot rule-out a potential non response bias whereby symptomatic individuals may have been more likely to respond to the survey than asymptomatic individuals. This bias would result in overestimating the prevalence of disease in this cohort. However, under the assumption that this response bias was independent of SHS exposure, the findings from the analyses assessing the association between SHS exposure and disease would not be affected. Second, the potential for recall bias exists which is an inherent limitation of self-reported medical surveys. Third, the survey design did not allow us to associate the timing of the exposure to the timing of the diagnosis for the index disease. Finally, SHS exposure is but one factor that may have contributed to our findings. Airplane cabin supply air is known to be contaminated with smoke and fumes containing pyrolyzed engine oil and/or hydraulic fluid which has been documented to cause respiratory complaints [19,20]. The air supply can also contain ozone gas in-flight and deicing fluid and/or exhaust fumes during ground operations [21-23]. Since 47 countries require that cabins be sprayed either in-flight or prior to boarding, pesticide exposure is another possible exposure and has been associated with respiratory illness [24,25]. FAs have also been identified as more likely to report infectious respiratory illnesses than the population of ground-based working women [26]. Notably, the per person ventilation rate in the aircraft is typically lower than in comparable ground-based environments which elevates bioeffluent levels and contaminants from cabin cleaners/deodorizers/offgassing cabin materials [27]. Finally, reduced barometric pressure in-flight and regular pressure changes may contribute to some respiratory conditions [28]. However, a previous study of smoking and non smoking airline flights observed a marked reduction in respirable particles and fewer symptoms such as ocular complaints, headaches, and fatigue in non-smoking flights [4]. This study would suggest that the elimination of SHS, with all other factors remaining the same, has a significant positive impact on the respiratory system.

## Conclusion

Despite these weaknesses, our study adds to the body of literature supporting a relationship between SHS and adverse health consequences among non smokers. Our data supports the need for continuing to implement clean indoor air policies to protect non smokers from the harmful effects of cigarette smoke.

## Abbreviations

SHS, Secondhand Smoke; FA, flight attendant; OR, odds ratio; CI, confidence interval

## Competing interests

The author(s) declare that they have no competing interests.

## Authors' contributions

RDH, ITC, DRS, and JOE designed and conducted the survey. DRS conducted the data analysis. RDH, ITC, DRS, JOE, and JM drafted the manuscript. All authors have read and commented on the manuscript.
